# Machine Learning for Recurrence Prediction of Gynecologic Cancers Using Lynch Syndrome-Related Screening Markers

**DOI:** 10.3390/cancers13225670

**Published:** 2021-11-12

**Authors:** Byung Wook Kim, Min Chul Choi, Min Kyu Kim, Jeong-Won Lee, Min Tae Kim, Joseph J. Noh, Hyun Park, Sang Geun Jung, Won Duk Joo, Seung Hun Song, Chan Lee

**Affiliations:** 1 Department of Information and Communication Engineering, Changwon National University, Changwon 51140, Korea; bwkim@changwon.ac.kr (B.W.K.); kkimlee@changwon.ac.kr (M.T.K.); 2 Comprehensive Gynecologic Cancer Center, CHA Bundang Medical Center, CHA University, Seongnam 13497, Gyeonggido, Korea; oursk79@cha.ac.kr (M.C.C.); p06162006@cha.ac.kr (H.P.); sgoncol@cha.ac.kr (S.G.J.); wdjoo@cha.ac.kr (W.D.J.); shsong@chamc.co.kr (S.H.S.); chanoncology@chamc.co.kr (C.L.); 3 Division of Gynecologic Oncology, Department of Obstetrics and Gynecology, Samsung Changwon Hospital, Sungkyunkwan University School of Medicine, Changwon 51353, Korea; 4 Gynecologic Cancer Center, Department of Obstetrics and Gynecology, Samsung Medical Center, Sungkyunkwan University School of Medicine, Seoul 06351, Korea; joseph.noh@samsung.com

**Keywords:** machine learning, immune checkpoint inhibitors, Lynch syndrome, recurrence

## Abstract

**Simple Summary:**

Recurrent patients with gynecologic cancer experience a difficult situation when using immune checkpoint inhibitors based on mismatch repair gene immunohistochemistry and microsatellite instability. Six machine learning algorithms were used to create predictive models with seven prospective features (four MMR IHC [*MLH1*, *MSH2*, *MSH6*, and *PMS2*], MSI, Age 60, and tumor size). The experimental results showed that the RF-based model performed best at predicting gynecologic cancer recurrence, with AUCs of 0.818 and 0.826 for the 5-fold cross-validation (CV) and 5-fold CV with 10 repetitions, respectively. This provides novel and baseline results of patients with recurrent gynecologic cancer using immune checkpoint inhibitors by using machine learning methods based on Lynch syndrome-related screening markers.

**Abstract:**

To support the implementation of genome-based precision medicine, we developed machine learning models that predict the recurrence of patients with gynecologic cancer in using immune checkpoint inhibitors (ICI) based on clinical and pathologic characteristics, including Lynch syndrome-related screening markers such as immunohistochemistry (IHC) and microsatellite instability (MSI) tests. To accomplish our goal, we reviewed the patient demographics, clinical data, and pathological results from their medical records. Then we identified seven potential characteristics (four MMR IHC [*MLH1, MSH2, MSH6,* and *PMS2*], MSI, Age 60, and tumor size). Following that, predictive models were built based on these variables using six machine learning algorithms: logistic regression (LR), support vector machine (SVM), naive Bayes (NB), random forest (RF), gradient boosting (GB), and extreme gradient boosting (EGB) (XGBoost). The experimental results showed that the RF-based model performed best at predicting gynecologic cancer recurrence, with AUCs of 0.818 and 0.826 for the 5-fold cross-validation (CV) and 5-fold CV with 10 repetitions, respectively. This study provides novel and baseline results about predicting the recurrence of gynecologic cancer in patients using ICI by using machine learning methods based on Lynch syndrome-related screening markers.

## 1. Introduction

Gynecologic cancers are among the emerging focuses of genome-based medicine [1]. They comprise cancers of the cervix, endometrium, and ovary. Furthermore, since publications about the high proportion of their target gene were reported during the data release of The Cancer Genome Atlas project [2,3,4], they have gradually drawn attention to precision treatment. The discovery of PARP inhibitors in BRCA mutation ovarian cancer was one of the publication’s promising results [5,6]. In addition to that therapy, germline gene-related hereditary cancer prevention surgeries have been used in the prevention of gynecologic cancer for decades [7]. Therefore, with the early adoption of new technology, target treatment, including the use of immune checkpoint inhibitors (ICIs), has proven effective in treating cancers of the cervix, endometrium, and ovary with or without available features, such as Lynch syndrome-screening-related biomarkers [8,9,10]. Furthermore, genome-related hereditary cancer syndrome has been well established in gynecologic clinical practice for a long time. Thus, in addition to Lynch syndrome, which is associated with the endometrium, the hereditary ovary and breast cancer syndrome, which affects these organs but also a small portion of the ovary, has received considerable attention for a long time Lynch syndrome is a hereditary genome-driven cancer syndrome affecting multiple organs, primarily the bowel and endometrium. This syndrome originates from mismatch repair (MMR) gene mutations as reported by Dr. Henry Lynch (1928–2019) [11]. As a result, prior to confirmative tests, several screening factors, such as family history, based on the Amsterdam criteria, in addition to tumor-based tests, such as immunohistochemistry (IHC) or microsatellite instability (MSI), have been proposed and are still used to treat patients around the world. These therapies are also used in clinical trials, even after attributable gene discovery and large-gene next-generation test adoption for cost-effectiveness [12].

Hence, new chemotherapy medicines, especially immunotherapy based on the target material have surprisingly been used in cancer treatments and patients with MMR-related cancer therapies, including those with Lynch syndrome with remarkable effects [13]. Recent studies show the predictive role of Lynch syndrome-screening factors in addition to IHC and MSI; other factors, such as PD-L1 are promising biomarkers [14]. However, inaccurate proven evidence exists among patients with recurrent gynecologic cancer based on MMR IHC and MSI receiving ICI.

Computer-based accurate models are another new technology in cancer medicine that is rapidly showing moderate importance, especially in the predictive and diagnostics field [15]. Gynecologic cancer has also embraced new technology in terms of predicting recurrence and developing prognosis models [16,17]. Furthermore, imaging study substitutions for diagnostic aids and lesion classification aids have recently been evaluated [18,19,20,21]. Among the most common Lynch syndrome-related cancer CRC studies are those using Lynch syndrome-screening markers and artificial intelligence, which has demonstrated that machine learning can predict MSI/dMMR with high accuracy in high-quality, curated datasets [22]. Recently, the possibility of machine learning models in ovary cancers has been demonstrated in terms of platinum sensitivity [23].

In this study, we explored various machine learning approaches for the prediction and classification of relapse in gynecologic cancers based on genome-based data. We also explore raw datasets to identify the seven important features (*MLH1, MSH2, MSH6, PMS2, MSI*, Age 60, and tumor size) for predicting the recurrence of cancer using one-hot encoding and Fisher’s exact tests. Furthermore, to provide a comparative analysis of the relapse of these gynecologic cancers, the performance of six machines, learning classifiers, LR, SVM, NB, random forest (RF), gradient boosting (GB), and extreme gradient boosting (XGBoost), was compared.

We hypothesized that by developing successful predictive models that can leverage genetic information, we can significantly expand the universe of cancer recurrence prediction, thereby creating more general and powerful machine learning tools. The experimental results showed that tree-based machine learning models, such as RF, GB, and XGBoost, learned high importance features from genetic data, resulting in a robust cancer recurrence prediction. Furthermore, the RF-based model predicted gynecologic cancer recurrence best, with AUCs of 0.818 and 0.826 for 5-fold CV and 5-fold CV with 10 repetitions, respectively. As a result, we created a suitable machine learning model for predicting the relapse of gynecologic cancers, allowing pathologists and clinicians to better diagnose cancers individually.

## 2. Materials and Methods

**Figure 1:**
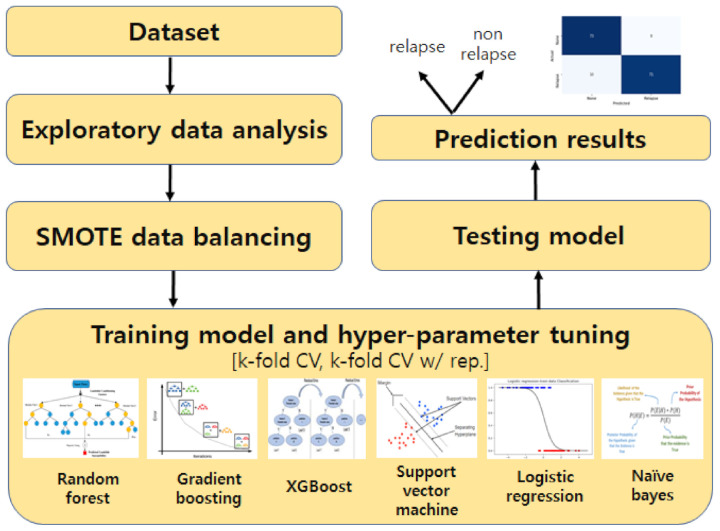
The simple architecture of the machine learning model to predict gynecologic cancer recurrence.

As mentioned earlier, this study investigated the impact of biomarkers, including Lynch syndrome screening markers, in classifying the performance for recurrent gynecological cancers using ICI. To achieve this goal, we followed experimental methodology, wherein we constructed datasets of various features that can affect the treatment and recurrence of cancer. Then, we looked at how certain features affected classification performance. Following preprocessing, six widely used classification models were trained on these datasets. Following that, the classifiers’ performance was assessed using various metrics for binary classification tasks and AUC. In the sections that follow, we will go over dataset selection, preprocessing, classification models used, and performance evaluation metrics. Figure 1 shows the overall block diagram of the machine learning model-based prediction of gynecologic cancer recurrence presented in this study.

### 2.1. Data Collection

We conducted a multicenter, retrospective study at three tertiary academic medical institutions in South Korea. Between January 2015 and December 2020, we reviewed the medical records of patients who were diagnosed with recurrent gynecological cancers using ICIs. Patient demographics, clinical data (age and tumor size based on imaging study results), and pathologic results, including MMR IHC staining for *MLH1* (MutL homolog 1)/*MSH2* (MutS Homolog 2)/*MSH6* (mutS homolog 6)/*PMS2* (*PMS1* Homolog 2), and MSI analysis, were all collected. As a result, 114 patients were included (Table 1). In addition, we added in Table 2 regarding MMR/MSI/Recur status of the study population

Among these patients, we reviewed serial radiological records of those diagnosed with recurrent or persistent gynecologic cancers who received at least one cycle of ICI treatment. Patients who were treated with ICIs underwent intravenous administration of 200 mg pembrolizumab every 3 weeks or 3 mg/kg nivolumab every 2 weeks until disease progression, unacceptable toxicity, or patient withdrawal. The institutional review board of each participating institution (CHA IRB 2020-12-034) approved the study protocol used in this research.

The clinicopathological characteristics of these patients are listed in Table 1. As stated, their median age was 54 years (range: 21–86). Among them, 41.2% (47/114) had an Eastern Cooperative Oncology Group (ECOG) performance status of ≤1, and 73.7% (84/114) had stage III or IV disease progression at initial diagnosis. Thus, eight tumor types were represented among the patients, with ovarian, cervical, endometrial, and uterine corpus cancers being the most common. Following that, PD-L1 expression was determined in 93 patients (81.6%), 65 (69.9%) of whom were PD-L1 positive. However, 38 (33.3%) of patients had tumors with abnormal MMR IHC (MMRd) and MSI (MSI high[h]: unstable MSI). Following that, specimens were labeled as MSI high if at least two allelic loci sizes shifted among the five microsatellite markers were examined. MSI-L was the abnormal one (low). MSS was the other classification (stable MSI). Both Bethesda (BAT25, BAT26, D5S346, D17S250, and D2S123) and Promega (NR21, NR24, NR27, BAT25, BAT26) markers used the MSI method. After DNA amplification using fluorescent-labeled primers, a panel of the five National Cancer Institute recommended microsatellite markers (BAT25, BAT26, D5S346, D17S250, and D2S123) or (NR21, NR24, NR27, BAT25, BAT26) were applied according to the manufacturer’s manual. IHC staining was performed on tumor specimens with 4 MMR genes (*MLH1*, *MSH2*, *MSH6*, and *PMS2*). Mouse monoclonal antibodies against *MLH1* (Novocastra Laboratories, Newcastle, UK), *MSH2* (Novocastra Laboratories), *MSH6* (BD Transduction Pharmingen, San Jose, CA, USA), and *PMS2* (Zytomed Systems, Berlin, Germany) were used. Bond–Max (Leica Biosystems, Newcastle, UK) immunostaining was carried out according to the manufacturer’s instructions. The slides were then reviewed and IHC data were evaluated by a gynecological pathologist. The intensity of the staining was graded as follows: negative, no discernible staining in tumor cells (loss: MMRd) total absence of nuclear staining in tumor each case had an adjacent normal stoma or infiltrating lymphocytes as an internal positive control; and positive, dark brown staining in tumor cells, completely obscuring the cytoplasm, nucleus, or membrane (intact: MMRp). The median sum of the target lesion size was 60 mm (range: 10–1230). Next, we classified the tumor burden according to tumor size based on the four classifications for machine learning (Size 1, 2, 3, and 4: <2, 2–5, 5–10, and ≥10 cm, respectively). The median number of lines before chemotherapy, including neoadjuvant chemotherapy, was two (range: 1–7), as observed. Nonetheless, pembrolizumab (88.6%, 101/114) and nivolumab (11.4%, 13/114) were the specific agents of ICIs administered. At the time of data cutoff on 28 February 2021, the median follow-up time was 4.9 months (range: 0.1–36.8). Eighty-five patients (74.6%) had stopped taking ICIs, most commonly due to disease progression. As a result, the patients received a median of four cycles (range: 1–40) of ICI-based chemotherapy. We first included 4 MMR IHC staining and 1 MSI analysis result. MMR IHC staining and MSI analysis results, also known by Lynch syndrome-screening markers, have been adopted in biomarkers in using ICI among several cancers. In addition, we discovered general risk factors in common among our diverse gynecologic cancers (43 ovaries, 37 cervices, 23 endometrium, 8 corpus, 2 GTT, and 1 vulvar cancer). As a sixth prospective feature, a 60-year-old age limit was adopted. Age is widely regarded as a significant factor in cancer prognosis. The definition of old age and its relationship to cancer and treatment differs depending on cancer and treatment type, but comorbidity situations are a more important indicator, and we could not find consistent age criteria based on evidence. But in Korea, the general retirement age by law is set as 60. It is based on common sense defining decreasing human function including patients with cancer and subdivided age classification like >50, 50–60, 60–70, >70 was not considered due to small numbers. However, in a future large study, other possible risk factors and subclassification will be used. Finally, measurable tumor size (Size 1, 2, 3, and 4: <2, 2–5, 5–10, and ≥10 cm) at recurrence was chosen as the 7th feature. Other probable prognostic factors (pathologic type and grade at first diagnosis, lymph node invasion, lymph vascular space invasion, initial clinical/surgical staging, variable tumor markers (CA 125, SCC, CEA, ß-hcg), previous platinum chemotherapy sensitivity, radiosensitivity) were not included due to specific characteristics of the study population (6 heterogeneous gynecologic cancers, recurrent cancer, ICI usage) and limited medical information from a retrospective study

### 2.2. Preprocessing and EDA

Among the features collected by the participating institutions, seven independent features that can affect cancer recurrence were considered and underwent exploratory analysis. The selected features for predicting cancer recurrence are *MLH1*, *MSH2*, *MSH6*, *PMS2*, MSI, Age 60, and size, which contain a mix of numeric and text datatypes (Table 3). The categorical data were then converted to vectors of real numbers to apply machine learning techniques to these qualitative data. For this analysis, we used one-hot encoding, which assigned each biomarker its own bit-encoded string (MLH1_intact: [1, 0, 0]; MLH1_loss: [0, 1, 0]). The input dataset for machine learning comprised 114 instances and 21 features.

To derive relevant information from the observed correlation between the relapse of tumors and the occurrence of genetic information, Fisher’s Exact Test was used as shown in Table 4. A *p*-value < 0.05 was considered statistically significant. As detected, we found a significant association between cancer occurrence, MLH1 intact (*p* = 0.0193), and PMS2 intact (*p* = 0.0147). However, the remaining features had a relatively weak correlation, which did not necessarily imply that there was no effect on gynecologic cancer recurrence. As a result, in this study, the machine learning model was built using all the obtained categorical data, as shown in Table 4.

### 2.3. Classification Models

We developed predictive models using selected features and evaluated the performance of these models. Next, we used six different widely used classifiers, which included LR, SVM, NB, RF, GB, and XGBoost. Below, we summarize these classification models:

(1) LR: An LR is a parametric classification model type and a particular form of the generalized linear model. This algorithm predicts a dependent data variable by analyzing the relationship between one or more existing independent variables.
(2) SVM: AN SVM is among the most popular machine learning models wherein a constraint finding the most stable discriminant boundary is added to, thereby forming a perceptron-based model. SVM defines a decision boundary with support vectors and classifies unclassified points by comparing them to the corresponding decision boundaries. The linear kernel is a fundamental function in SVM; however, the radial basis function (RBF) kernel is a popular kernel function used in a variety of kernelized learning algorithms.
(3) NB: The NB algorithm is a probabilistic machine learning algorithm that uses the Bayes theorem. Under the naive assumption that all events are independent, it makes probabilistic predictions by inferring posterior probabilities from prior probabilities. The NB classifier has the advantage of being very efficient to train and requiring a small amount of training data to estimate the classification parameters.
(4) RF: The RF algorithm can predict an output variable based on a majority vote after generating multiple decision trees. As a result, a decision tree can schematize decision rules and their outcomes in the form of a tree structure. The data is then split into multiples by a tree-based model based on specific cutoff values of the feature values. The RF can then improve on the performance of a single tree algorithm by combining decision trees and bagging, where various samples can be extracted from the training set using bootstrap.
(5) GB: Alternatively, the GB algorithm creates a more accurate and stronger learner by combining a simple and weak decision tree. Although the accuracy of the weak tree model reveals a flaw in prediction error, it can be compensated for using the second model. As a result, combining these successively weak tree models yields a more accurate model than the first. In GB, the residual is fitted with a weak tree model, and the predicted value is then updated by adding the predicted residual to the previous prediction.
(6) XGBoost: XGBoost is another model that has been in the spotlight recently in tree-based ensemble learning. Although XGBoost is based on GB, it can overcome GB’s drawbacks, such as slow execution time and a lack of overregulation. XGBoost, in particular, can learn histories in a parallel CPU environment. Therefore, it can complete training faster than the existing GB model.

### 2.4. Performance Evaluation

For a machine learning model to be robust, it is required to use many observations to reach acceptable performance levels. This gynecologic cancer diagnosis-based machine learning study, on the other hand, has a small sample size. Furthermore, when using the machine learning approach, the compositions of the training and test sets differ because the dataset was randomly divided. According to our observations, the results varied greatly depending on how datasets were divided into training and test sets, particularly with a small sample size.

Note that no fixed rule exists for separating training and testing data sets, and the common split ratio is 80%:20% for train and test sets, respectively. However, the size of our study population’s dataset is limited. As a result, we divided the oversampled data set 85 percent for training and 15% for testing. We also used the training set to learn and tune the machine learning algorithm’s hyperparameters. Furthermore, to select the best hyperparameters, we validated our model on the validation dataset to obtain unbiased estimate results. We performed 5-fold CV by dividing the training set up into five equal-sized portions. Then, to examine the generalized performance of the proposed scheme and minimize the effect of data composition by a random training test set split, the split was subsequently repeated 10 times by changing the random state numbers and providing average results.

Performance measures play a critical role in machine learning model development, hyperparameter tuning, and evaluation. Various performance measures were used in this study to evaluate different machine learning algorithms for classification. In this study, false-positive rates (FPR), true positive rates (TPR), specificity, positive predictive values (PPV), negative predictive values (NPV), accuracy, and F-scores have widely used metrics for evaluating classifier performance for a specific threshold. At certain threshold values, these metrics can be calculated. Receiver operating characteristic (ROC) curves are also used to represent a tradeoff between different classifiers in a variety of scenarios. As a result, rather than being dependent on a specific threshold, the area under the ROC curve (also known as AUC) can be used to provide a summary of how well a classifier performs at various threshold settings for the classification task. In this case, the larger the AUC area, the more accurate the classifier model. Moreover, AUC scores can assess the quality of classification more reliably compared with accuracy in various situations based on the given dataset.

Furthermore, ROC curves summarize classifier performances over several FPRs (or fallout) and TPRs (or recall) for every possible cutoff. The TPR sensitivity or recall is thus defined as the percentage of recurrence of cancer that is correctly identified as the patients with cancer of relapse:

(1) $$\mathrm{Recall}=\mathrm{Sensitivity}=\mathrm{TPR}=\;\frac{\mathrm{TP}}{\;\mathrm{TP}\;+\;\mathrm{FN}},$$

However, the specificity or true negative rate (TNR) is defined as the percentage of cancer recurrence that is correctly identified as the patients with cancer of relapse:

(2) $$\mathrm{Specificity}=\mathrm{TNR}=\frac{\mathrm{TN}}{\mathrm{TN}\;+\;\mathrm{FP}},$$

In contrast, the FPR or fallout is defined as 1-Specificity. For all possible cutoff values, ROC curves are drawn by plotting the sensitivity (TPR) along the Y-axis and the corresponding 1-specificity (FPR) along the X-axis. It is the set of all ordered pairs in mathematics (FPR, TPR). Alternatively, PPV (or precision) is the probability that a positive observation is truly positive, whereas NPV is the probability that a negative observation is truly negative.

## 3. Results

In the following sections, the experimental results obtained are presented for the classification models using the collected gynecologic cancer datasets. Following that, we created a binary classification task for the six machine learning classifiers to perform on the experimental dataset. We used Python 3.9 with the Scikit-learn library 1.0 to create machine learning models, which provides a wide range of machine learning techniques. The experiments were carried out on a computer with a 3.6 GHz CPU, a Core i9 processor, and 64.0 GB of memory. Because the number of cancer relapse cases was significantly lower than the number of cases in the other class, the raw dataset obtained was unbalanced. Learning from imbalanced data affects the training process of classifiers, leading to an unfavorable bias toward the majority class and resulting in low accuracy for the minority class. To build the balanced dataset, the Synthetic Minority Oversampling Technique (SMOTE) is used, which generates new synthetic training data for the minority class by adding random values to some features of the original training data and providing new data that is as close to the original as possible. For each sample in the minority class, synthetic training data are generated by randomly selecting one or more of the k-nearest neighbors. As a result of SMOTE, 162 instances were obtained, with 81 cases of recurrence and no recurrence, respectively.

By dividing the oversampled data set into 85% for training and 15% for testing, training and parameter tuning of the machine learning model were conducted using training data. Then, for RF, GB, XGB, SVM, NB, and LR, we tuned hyperparameters to get the optimal hyperparameter combination with the highest means of validation for AUC values. The best parameters were then identified using the grid search method, and the model’s performance was validated using two CV methods. In addition to the standard 5-fold CV without repetition, a 5-fold CV with 10 repetitions was performed and compared. CV was repeated 10 times with data shuffling for each combination of hyperparameters in CV with 10 repetitions. Furthermore, the AUC score was used as the model’s performance evaluation metric. Table 5 summarizes six classification models and their hyperparameter ranges for using the grid search approach.

### 3.1. Classification Results

To validate the prediction performance of our machine learning algorithms, a ROC curve analysis was employed to determine the AUC. The performance of the six classification models—RF, GB, XGBoost, NB, LR, and SVM (linear kernel, RBF kernel)—when trained on the dataset is presented in Table 6 for the AUC score. Depending on which samples comprised the training or test set, AUCs in CV, training, and testing varied widely. Thus, the split was repeated 10 times by changing random state numbers, which provided the average AUC results.

**Figure 2:**
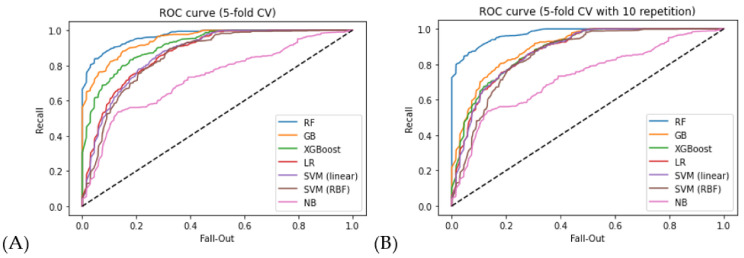
Seven models’ ROC curves for training datasets: (**A**) 5-fold CV; (**B**) 5-fold CV with 10 repetitions.

The six models’ ROC curves for training datasets created from the two CV methods are shown in Figure 2. The ROC curve is generated by plotting the TPR against the FPR at various threshold settings, as explained in Section 2.4. The steeper the curve toward the upper left corner, the better the classifier’s ability to distinguish between classes. When comparing different ROC curves, especially when they intersect, the area under the ROC curve can be used to evaluate models. Among the six models, the RF-based model performed the best in identifying cases of recurrent cancer across all CV methods. Moreover, other tree-based approaches, such as GB and XGBoost, present robust performances in classifying patients with recurrent cancer.

**Figure 3:**
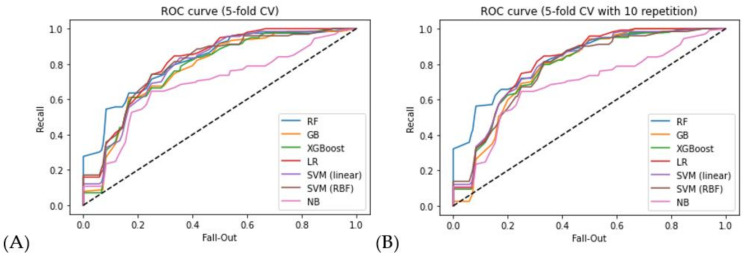
Seven models’ ROC curves for test datasets: (**A**) 5-fold CV; (**B**) 5-fold CV with repetitions.

Figure 3 further depicts ROC curves for test datasets using seven machine learning algorithms. Similar to the results of the training datasets, the RF-based model outperformed the other seven candidates for all CV methods. It can also be seen here that the LR technique outperformed the RF technique. However, when compared to other tree-based methods, LR did not perform well on the training data set. As a result, this result incorporated the impact of fluctuations caused by the absence of the test data set. It can be observed as well that the tree-based method showed satisfactory cancer recurrence classification performance even for the test data.

Table 6 shows their classification results for the test datasets in the two CV methods. Decision tree-based classifiers, including RF, GB, and XGBoost, worked well in the cancer recurrence classification task because their AUC values were approximately 0.8. Among these candidates, the RF algorithm had the highest AUC score of 0.818 for 5-fold CV and 0.826 for 5-fold CV with 10 repetitions, respectively. Other tree-based schemes, such as GB and XGBoost, performed similarly to RF in terms of AUC scores for various CV methods. Nevertheless, in all cases, a performance difference was observed between the 5-fold CV and 5-fold CV iteration techniques, which is caused by the small number of instances in the dataset.

### 3.2. Classification Performance on Tree-Based Approaches

**Figure 4:**
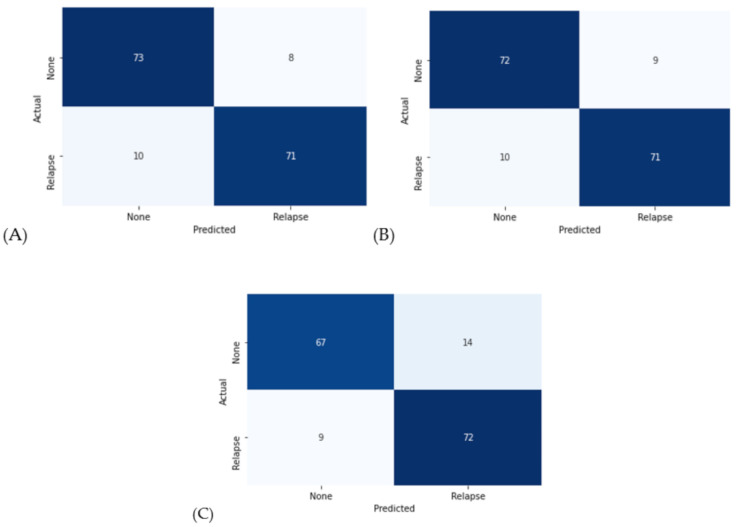
Confusion matrices with the tree-based machine learning approach: (**A**) Random forest; (**B**) Gradient boosting; (C) XGBoost.

Figure 4 displays confusion matrices of the tree-based approaches, including RF, GB, and XGB. A confusion matrix is a specific table layout that allows for the visualization of algorithm performance, and it is most used in classification performance. Each row of the matrix represents data samples from an actual class, while each column represents samples from a predicted class. This provides us with a comprehensive picture of how well our classification model is performing and the types of errors it is making. Because the number of test dataset instances was too small, confusion matrices were expressed for the entire dataset. Table 7 displays their classification results based on popular performance metrics. Furthermore, in terms of accuracy and sensitivity (or recall), the RF algorithm showed the best performance of 88.88% and 90.12%, respectively. However, except for the sensitivity, the GB algorithm also showed similar performances to the RF. In contrast, for specificity and PPV, XGBoost outperformed other tree-based schemes.

## 4. Discussion

Machine learning technology enables the development of high-performance predictive models and can be used in various cancer diagnostics and therapeutics. To the best of our knowledge, this is the first study to assess machine learning for patients with recurrent gynecologic cancer under ICI using Lynch syndrome-related screening markers such as MMR IHC and MSI. However, because this gynecologic cancer diagnosis-based machine learning study was conducted with a limited number of samples, when clinicians use the machine learning approach, the compositions of the training and test sets differ because the dataset was randomly split. When the RF algorithm was used to predict cancer recurrence, it achieved accuracy, sensitivity, and specificity of 88.88 percent, 90.12 percent, and 87.65 percent, respectively. For other tree-based schemes such as GB and XGBoost, the classification results outperform SVM, LR, and NB in terms of various performance metrics.

The current study has several limitations. The first issue is the small size of the study population. However, recurrent gynecologic cancer under ICI is in a difficult situation, and previous reports show a limited but durable and sometimes striking repose with specific genomic [24]. That could be the reason for using machine learning in this machine. The design of a large prospective trial is centered on combining Lynch syndrome-related screening markers with previous PD L1 or other clinical markers (tumor burden or size) or performance score-like age. Our study’s seven figures are unique in that they combine clinical and genomic information in this rare population. Second, no specific criteria were used to select ICI between pembrolizumab and nivolumab. Therefore, there was no specific better recommendation of ICI regimen for this specific population [25]. After several ICI clinical combination trials may have a better solution in the future. Third, there is a scarcity of incomplete follow-up data and periods for predicting disease responses and recurrence. This study population has a low-performance status (ECOG 2–4 58.8%), a high initial advanced stage (II/IV 73.7%) and has been heavily treated (previous chemotherapy was 40.4%). We conducted our research based on short-term follow-up due to the poorer performance status, most advanced stage population, and heavily treated history. Pathologic markers, on the other hand, are not included. Because of the high initial advanced status (II/IV 73.7%), only half of the full staging operations (57/114) were completed. Imaging studies such as CT, MRI, and/or PET-CT were used to assess staging. The majority of IHC and MSI were performed on biopsy specimens. This is our study limitation. Initial surgical specimen tumor factors like LVSI (lymph vascular space invasion), LN meta were not considered in this study population. Instead of clinical lymph node invasion or surgical LVSI information, we added LN meta sized at recurrence and divided it by 4 groups (Size 1, 2, 3, and 4: <2, 2–5, 5–10, and ≥10 cm) based on imaging result. If data obtained through 15 [High-grade serous carcinoma (*n* = 32), Squamous cell carcinoma (*n* = 23), Endometrioid adenocarcinoma (*n* = 22), Clear cell carcinoma (*n* = 7), Mucinous carcinoma (*n* = 6), Leiomyosarcoma (*n* = 5), Mixed adenocarcinoma (*n* = 5), Adenosquamous carcinoma (*n* = 4), Neuroendocrine carcinoma (*n* = 2), Carcinosarcoma (*n* = 2), Endometrial stromal sarcoma (*n* = 2), Gestational thromboplastic tumor (*n* = 1), Mesonephric adenocarcinoma (*n* = 1), Endocervical adenocarcinoma (*n* = 1), Germ cell tumor (*n* = 1)] pathologic type is added to existing data, an attempt can be made to increase the accuracy of cancer recurrence prediction thanks to the diversity of variables used as inputs for machine learning. In general, if the number of variables is increased for implementing the machine learning algorithm, the number of data samples should also increase. However, since the number of data samples used in this study is small, the performance evaluation of cancer recurrence prediction was conducted without considering pathology variables. When a larger number of patient data samples are provided in the future, we will conduct a study to further increase the probability of predicting cancer recurrence by including pathology data.

In a previous study on ovarian carcinoma prediction [21], a machine learning model, which can predict platinum sensitivity with high-grade serous ovarian carcinoma, obtains an LR model with an AUC of 0.741 performed best at identifying platinum-resistant cases. The LR model is one of the most basic models in machine learning, and it cannot solve nonlinear problems because it has a linear decision surface. The performance of the LR-based model for ovarian carcinoma prediction [21] was reported to be comparable or superior to that of the RF, SVM, or DNN. This means that the ovarian carcinoma prediction problem is linearly separable, which is uncommon in real-world scenarios. However, when it came to the relationship between gynecological cancer recurrence and genetic factors, this study found that the tree-based machine learning method outperformed LR. This means that the problem of gynecological cancer recurrence is close to a nonlinear problem, and a more powerful and complex prediction method is required.

Clinicians and patients are curious about the progression of cancer but statistical analysis and individual courses are different according to complex pathogenic situations affecting a patient’s illness with the advance of modern technology including diagnostic and therapeutic modalities [26]. In addition to conventional scarce epidemiology, low incidence rare diseases such as Lynch syndrome may necessitate special consideration to improve accuracy and wise decision-making for patients [27]. Because there are many ambiguities and uncertainties in modern pathology and radiology, there should be an additional system to support and assist clinicians in making cancer-related decisions [28]. Commercially available information technology is assisting in the efficient enrollment of clinical trials [29]. This research could be crucial in the future for rare cancer population standard treatment and clinical trial development. Patients with recurrent cancer face a slightly different situation than those who were initially diagnosed. The goals of recurrent patients differ depending on the recurrent location sites and performance status. ICI has rapidly contributed to cancer treatment also in patients with gynecologic cancer p but prediction parameters are not solely dependent on specific factors [30]. As of now, current biomarkers such as PD-L1 IHC are expected to be not adequate for response under ICIs [31]. Although there are many maps to cancer treatments, there is not yet an accurate navigator. Our machine-learning-based approach holds promise for predicting the recurrence of gynecological cancers based on genetic factors, thereby reducing over- and under-treatment.

## 5. Conclusions

This study developed machine learning models to assist clinicians in efficiently treating gynecologic cancer recurrence with ICIs from genome-based data. Using one-hot encoding and Fisher’s-exact tests, we identified important features in the given datasets. Predictive models were developed with selected features using six machine learning algorithms (LR, SVM, NB, RF, GB, and XGBoost), and their performances were compared in terms of various classification evaluation metrics and AUC. Because of the small size of the dataset used in this study, we only performed 10 random splits of the training and test datasets to provide average AUC values. The model’s performance was then assessed using the 5-fold CV and 5-fold CV with 10 repetitions methods. The experimental results demonstrated that the RF model had the best prediction performance in gynecologic cancer recurrence, with AUCs of 0.818 and 0.826 for 5-fold CV and 5-fold CV with 10 repetitions, respectively. Our findings can help improve the efficiency and accuracy in treating and predicting treatment response in this rare and specific population.

## Figures and Tables

**Table 1:** Baseline characteristics of the patients treated with immune checkpoint inhibitors (*n* = 114).

Age, Years	Variable
Median (range)	54 (21–86)
ECOG performance status	
0–1	47 (41.2%)
2–4	67 (58.8%)
FIGO stage at diagnosis	
I/II	22 (19.3%)
III/IV	84 (73.7%)
N/A	8 (7.0%)
Origin of cancer	
Cervix	37 (32.5%)
Vulvar	1 (0.9%)
Ovary/Peritoneum/fallopian tube	43 (37.7%)
Endometrium	23 (20.2%)
Uterine corpus	8 (7.0%)
Gestational trophoblast	2 (1.8%)
PD-L1 expression *	
≥1	65 (57.1%)
<1	28 (24.6%)
N/A	21 (18.4%)
MMRd	26 (22.8%)
MMRp	88 (77.2%)
MSI-H	11 (9.6%)
MSI-L	6 (5.2%)
MSS	97 (85.1)
MMRd or MSI-H	38 (33.3%)
MMRp or MSS	76 (66.7%)
Target lesion size, mm ^#^	
Median, range	60 (10~1230)
Number of previous lines of chemotherapy	
1	29 (25.4%)
2	39 (34.2%)
3	18 (15.8%)
4	15 (13.2%)
≥5	13 (11.4%)
Type of immune checkpoint inhibitor	
Pembrolizumab	101 (88.6%)
Nivolumab	13 (11.4%)

ECOG, Eastern Cooperative Oncology Group; FIGO, International Federation of Gynecology and Obstetrics; N/A, nonavailable; PD-L1, programmed death-ligand 1; MMRd, mismatch repair deficiency; MSI-H, microsatellite high; MMRp, mismatch repair proficiency; MSS, microsatellite stable. * Determined by either the tumor proportion score or the combined positive score. ^#^ Sum of the diameters of the target lesions.

**Table 2:** MMR/MSI/Recur status of the study population.

Origin	MMRd	MMRp	MSI-H	MSI-L	MSS	Recur
Cervix	7 (18.9%)	30	3 (8.1%)	0	34	25 (67.6%)
Ovary	7 (16.3%)	36	2 (4.7%)	5	36	36 (83.7%)
Endometrium	9 (39.1%)	14	4 (17.4%)	1	18	16 (69.6)
Others	3 (33.3%)	8	4 (18.2%)	0	9	4 (36.4%)
Total	26 (22.8%)	88	11 (9.6%)	6 (5.2%)	97	81 (71.1%)

Ovary: Ovary/Peritoneum/fallopian tube; Others: Vulvar/Uterine corpus/Gestational trophoblast.

**Table 3:** Dataset descriptions.

Attributes Notation	Attributes	Raw Data Type	One-Hot Encoded Attributes
*MLH1*	MutL homolog 1	Text	MLH1_intact
			MLH1_loss
			MLH1_none
*MSH2*	MutS homolog 2	Text	MSH2_intact
			MSH2_loss
			MSH2_none
*MSH6*	MutS homolog 6	Text	MSH2_intact
			MSH6_loss
			MSH6_none
*PMS2*	PMS1 homolog 2	Text	PMS2_intact
			PMS2_loss
			PMS2_none
MSI	Microsatellite instability	Text	MSI_high
			MSI_low
			MSI_stable
Age 60	Age greater/less than 60	Numeric	Age60
Size	Tumor size	Numeric	Size_1
			Size_2
			Size_3
			Size_4

**Table 4:** Associations between recurrence of gynecologic cancers and categorical feature set based on Fisher’s exact test.

Attributes	Fisher Odd Ratio	Attributes	Fisher Odd Ratio
	(*p*-Value)		(*p*-Value)
*MLH1* intact	3.1233	PMS2 intact	3.6571
	(0.0193)		(0.0147)
*MLH1* loss	0.8	PMS2 loss	0.9062
	(0.7167)		(1.0)
*MSH2* intact	1.84	MSI high	0.448
	(0.2079)		(0.2920)
*MSH2* loss	1.6623	MSI low	2.1052
	(1.0)		(0.6706)
*MSH6* intact	1.5180	MSI stable	0.7169
	(0.4018)		(0.5216)
*MSH6* loss	1.7846	Age 60	1.2606
	(0.4221)		(0.6610)
Size 1	1.0706	Size 2	0.8172
	(1.0)		(0.8107)
Size 3	1.2392	Size 4	0.9455
	(0.8171)		(1.0)

**Table 5:** Classification models and hyperparameter ranges for the grid search.

Classification Model	Hyperparameter Ranges
Random forest Gradient boosting XGBoost	Maximum depth of tree = [1, 3, 5, 10, 15, 20, 25, 30] Number of estimators = [10, 50, 100, 200, 500, 1000, 1500, 2000] Learning rate = [1, 0.1, 0.01, 0.001, 0.0001, 0.00001]
Support vector machine	C = [0.001, 0.01, 0.1, 1, 10, 100, 1000, 10,000] Kernel = [“linear,” “rbf”] Gamma = [0.5, 0.1, 0.01, 0.001, 0.0001]
Logistic regression	C = [0.001, 0.01, 0.1, 1, 10, 100, 1000, 10,000]

**Table 6:** Classification results.

ML Algorithms	5-Fold CV	5-Fold CV Rep. 10
RF (train)	0.972	0.978
RF (test)	0.818	0.826
GB (train)	0.952	0.900
GB (test)	0.779	0.782
XGBoost (train)	0.917	0.883
XGBoost (test)	0.767	0.778
LR (train)	0.872	0.876
LR (test)	0.803	0.801
Linear SVC (train)	0.871	0.875
Linear SVC (test)	0.782	0.792
Kernel SVC (train)	0.729	0.729
Kernel SVC (test)	0.675	0.675
NB (train)	0.855	0.855
NB (test)	0.791	0.773

rep, repetition.

**Table 7:** Classification results.

ML Algorithms	Accuracy (%)	Sensitivity (%)	Specificity (%)	PPV (%)	NPV (%)
Random forest	88.88	90.12	87.65	87.95	89.87
Gradient boosting	88.27	88.88	87.65	87.8	88.75
XGBoost	85.8	82.71	88.88	88.15	83.72

## Data Availability

Data are contained within the article.
